# Serum HE4: An Independent Prognostic Factor in Non-Small Cell Lung Cancer

**DOI:** 10.1371/journal.pone.0128836

**Published:** 2015-06-01

**Authors:** Pierre-Jean Lamy, Carine Plassot, Jean-Louis Pujol

**Affiliations:** 1 Department of Biology and Oncogenetics, Montpellier Cancer Institute, Montpellier, France; 2 ICM-Biobank, Regional Cancer Center, Montpellier, France; 3 Department of Statistics and Epidemiology, Institute for Clinical Research, University Montpellier I, Montpellier, France; 4 Thoracic Oncology Unit, Hospital University Center of Montpellier, Montpellier France; University of Algarve, PORTUGAL

## Abstract

Human epididymis secretory protein 4 (HE4) is a secreted glycosylated protein encoded by the WAP four-disulfide core domain 2 (*WFDC2*) gene, located on a chromosome 20 segment that is frequently amplified in many cancers. This study aimed at determining serum HE4 prognostic value in non-small cell lung cancer (NSCLC), following the REMARK guidelines. Serum samples from 346 consecutive patients with histologically proven and previously untreated NSCLC and 41 patients with benign pulmonary disease were collected at the Montpellier-Nimes Academic Hospital. Work-up investigations performed to determine the disease characteristics and treatment algorithms were congruent with international guidelines. HE4 levels in serum were measured with an ELISA test (Fujirebio Diagnostics) that uses two monoclonal antibodies, 2H5 and 3D8, against the C-WFDC domain of HE4. The area under the ROC curve (i.e., overall ability of HE4 to discriminate between controls and patients) was 0.78 (95% confidence interval [CI], 0.738–0.821; z test *P* <0.0001). Serum HE4 levels were significantly higher in patients with worse performance status, advanced TNM stage and positive nodal status. In the Cox model, overall survival was shorter in patients with high pretreatment serum HE4 (above 140 pmol/L) than in patients with serum H4 level ≤ 140 pmol/L [median survival: 17.7 weeks (95% CI, 11.9 to 24.9) and 46.4 weeks (95% CI, 38.6 to 56.3), respectively; hazard ratio: 1.48 (95% CI, 1.12 to 1.95) for high HE4; adjusted *P* = 0.0057]. High serum HE4 level at diagnosis is an independent determinant of poor prognosis in NSCLC.

## Introduction

The identification of prognostic determinants of non-small cell lung cancer (NSCLC) is an important goal in both clinical trials and routine practice [1,2,3,4]. In clinical trials, prognostic co-variables must be taken into account in survival analyses. For instance, in a randomized trial, the statement that a difference in survival is related to the effect of the treatment under study must be supported by a proportional hazards model demonstrating that this effect does not depend on well-known prognostic determinants, such as disease stage or performance status [5,6]. In routine practice, therapeutic decision-making can be influenced by prognostic variables [4]. The most widely-accepted prognostic determinants in NSCLC are disease stage, nodal status and performance status [7]. Several other features, particularly male gender, age, non-squamous histology, have been variously reported as negative prognostic factors [2,8]. Recently, molecular biomarkers, such as EGFR mutations and ALK translocations, have been introduced as theragnostic markers of lung adenocarcinoma.

Human epididymis secretory protein 4 (HE4) is a secreted glycosylated protein belonging to the WFDC (previously named WAP) family. WAP four-disulfide core domain 2 (*WFDC2*), the gene encoding HE4, is located on chromosome 20, in a segment frequently amplified in many cancers (breast, ovarian, colon, pancreas, and lungs)[9]. Other proteins in this family include Secretory Leukocyte Peptidase Inhibitor, Elafin, and PS20 [10]. Members of the WFDC family are characterized by the presence of one or more WFDC domains of approximately 50 amino acids in length that contain eight highly conserved cysteine residues linked by four disulfide bonds [11]. HE4 is a protease inhibitor that was identified in the epithelium of the epididymis and is involved in sperm maturation [12]. The protein shows characteristics of a secretory protein, with a signal peptide followed by a small (approximately 10-kDa), acidic (pI 4.3), and cysteine-rich polypeptide [12]. Its role in other tissues remains unclear. It has been suggested that it might be involved in the innate immunity defenses of the respiratory tract, nasal and oral cavities and in the development of lung adenocarcinoma [13]. Moreover, HE4 is over-expressed in ovarian cancer, particularly in serous, clear cell and endometroid epithelial ovarian carcinomas [14], and is secreted early in the serum of patients with ovarian cancer [15]. Several attempts have been done to characterize ovarian cancer using multi-parametric models of gene expression, including HE4 mRNA [16] [17]. Many publications have shown that the serum levels of HE4 and CA125 can be used for the early detection of ovarian cancer recurrence and to classify patients with a pelvic mass as at high or low risk of ovarian malignancy [18,19]. Its specificity is higher than that of CA125, especially in early stage disease and in premenopausal women [20]. Serum HE4 level is correlated with tumor stage [21], age and smoking status [22]. Importantly, it is not elevated in benign gynecological conditions and in endometriosis [23]. However, HE4 is not ovarian cancer-specific. Indeed, *WFDC2* is strongly expressed in normal human trachea and salivary glands and, to a lesser extent, in lung, prostate, pituitary gland, thyroid and kidney. Moderate to high levels have also been detected in lung adenocarcinoma and, occasionally, in breast, transitional cell and pancreatic carcinomas [24]. Particularly, HE4 is expressed in most lung adenocarcinomas and in a significant number of squamous, small cell and large cell carcinomas of the lung, suggesting that it could be used as a diagnostic and/or prognostic factor to refine the standard pathologic analysis [13]. Indeed, in lung adenocarcinoma, nodal status and HE4 expression are independent prognostic factors of disease-free and overall survival [25]. Moreover, high serum and pleural effusion concentrations of HE4 were previously observed in NSCLC [26,27] and Three studies suggested that HE4 could be a potential diagnostic and prognostic marker in NSCLC [28,29,30].

The aim of our study was thus to determine the diagnostic and prognostic value of HE4 serum level using 346 samples from a serum biobank dedicated to the validation of biomarkers in lung cancer and following the REMARK (Reporting Recommendations for Tumor Marker Prognostic Studies) guidelines [31].

## Materials and Methods

### Patients

Serum samples from 346 consecutive patients with NSCLC referred to the Montpellier—Nîmes University Hospital, France, between January 1995 and December 1997 were used for this study. These samples are part of a large biobank (Biobank number BB-0033-00059-Certified AFNOR 96–900) that was started in 1990 to collect serum samples from patients with NSCLC with the goal of determining prospectively the prognostic impact of new serum tumor markers. Specifically, the biobank protocol defined the clinical variables to be recorded, the eligibility criteria and the statistical methods to be used. The methodology did not change over time. All biobank samples are associated with comprehensive clinical data that were prospectively recorded and samples are stored at—180°C (in triplicate). However, this study was retrospective because the decision to test HE4 has been taken only recently. This study was reviewed and approved by the ICM Institutional Review Board called “Comité d’Organisation de la Recherche Translationnelle” (CORT). According to the French regulation, the consent for secondary use of human biological material is under a legal regime of “non-opposition” (opt-out): After information for new use from researchers, human biological samples can be used except in case of opposition from the donor. Then, inform consent was not obtained from the patients but patient records/information was anonymized and de-identified prior to analysis.

Only serum samples from patients with NSCLC histologically proven and previously untreated NSCLC were used for this study. NSCLC cancers were classified according to the WHO histological classification; however, the last revision concerning the new taxonomy of adenocarcinomas was not taken into account [32] and adenocarcinoma was considered as a generic sub-histologic type. Performance status was estimated using the Eastern Cooperative Oncology Group (ECOG) score and the percentage of weight loss during the previous four months was recorded. Staging was carried out according to the *Union for International Cancer Control* (UICC) tumor node metastasis (TNM) classification in use at the time of diagnosis [33] and the American Thoracic Society map of regional pulmonary nodes [34]. The following investigations were carried out: clinical examination, standard chest roentgenography, computed tomography (CT) scan of chest, upper abdomen and brain, fiberoptic bronchoscopy, liver sonography and bone scintigraphy. Mediastinoscopy was performed to establish the node status in patients with non-metastatic NSCLC, but evidence of mediastinal lymph node enlargement on the chest CT images.

### Controls

Serum samples were collected from 41 consecutive patients with non-malignant pulmonary diseases (infectious diseases, chronic obstructive pulmonary diseases and miscellaneous). Patients in the control group have similar median age (60 years) and sex ration than patients in the lung cancer group.

### Treatment

A medical panel composed of thoracic surgeons, chest physicians, radiologists, radiotherapists and medical oncologists discussed each patient’s medical record. Patients with stage I-II NSCLC or resectable IIIa disease underwent surgery with the aim of achieving complete resection. Patients with performance status 2 and distant metastases (stage IV) or gross mediastinal involvement (stage IIIb and unresectable stage IIIa tumor) were eligible for enrolment in one of the cisplatin-based chemotherapy trials (cisplatin and a third generation drug) conducted at the Montpellier University Hospital. Concurrent radiotherapy was proposed to patients with locally advanced disease [5]. Active supportive care, including palliative radiation therapy when needed, was given to patients with advanced stage and poor performance status. Treatment was decided based on clinical and routine biological findings and without knowledge of the level of serum markers. Hence, treatment was not considered as a prognostic variable in this study.

### Laboratory methods

A blood sample was collected from each patient at diagnosis and serum was separated and stored at -180°C until tested. HE4 was measured by using the commercial EIA method (Fujirebio Diagnostics, Malvern, PA). This test is intended for use with serum and is a solid-phase, non-competitive immunoassay based on the direct sandwich technique using two mouse monoclonal antibodies, 2H5 and 3D8, against two epitopes in the C-WFDC domain of HE4. HE4 present in calibrators or samples is adsorbed to streptavidin-coated microstrips by the biotinylated anti-HE4 antibody 2H5 during the incubation. Strips are then washed and incubated with HRP-labeled anti-HE4 mAB 3D8. After washing, a buffered substrate/chromogen reagent (hydrogen peroxide and 3, 3’, 5, 5’ tetra-methyl-benzidine) is added to each well. During the enzyme reaction a blue color will develop if the antigen is present. The color intensity is proportional to the amount of HE4 in the sample and is determined using a microplate spectrophotometer at 405 nm after addition of the Stop Solution. Calibration curves (Cubic Spline) were constructed for each assay by plotting the absorbance value versus the concentration of each calibrator. HE4 concentrations in patients’ samples were then calculated based on the calibration curve. The HE4 EIA assay measures H4 concentrations between 15 and 900 pM. We previously validated the test in our laboratory following the COFRAC LAB GTA 04 methodology. The limit of detection was 4 pmol/L and the limit of quantification was 8 pmol/L. The intra- and inter-assay coefficients of variation were lower than 10%. Serum neuron-specific enolase (NSE) was measured by using the solid phase two-site immunoradiometric assay ELSA NSE (CIS bio International, Gif-sur-Yvette, France) and serum CYFRA 21–1 was determined with the IRMA method (CIS bio International, Gif-sur-Yvette, France), as previously described [35]. Other biological variables tested in this study were measured before any treatment and concomitantly to the titrations of tumor markers (but for HE4) in a laboratory that implemented good laboratory practice. The upper limit of normal values was 10 000/ml for leukocytes. The lower limits of normal values were 32 g/ l for albumin and 135 mmol/L for serum sodium.

### Statistical analysis

Receiver Operating Characteristic (ROC) curves were constructed using both patients’ and controls’ serum marker levels in an attempt to establish the sensitivity—specificity relationship for each marker. The areas under the ROC curves were calculated [28]. ROC curve analysis was carried out using the Medcalc statistical software (Ostend, Belgium). The sensitivity-specificity relationship was determined using the Youden's index J, which is the difference between the true positive rate and the false positive rate. The normal (Gaussian) distribution of serum HE4 levels was tested using the non-parametric Shapiro—Wilk test for the equality of continuous, one-dimensional variables. For HE4 in the tested populations, the test was significant, thereby rejecting the H0 hypothesis (i.e., demonstrating that serum HE4 level was not distributed according to the Gaussian law). Therefore, to analyze the distribution of tumor markers in subsets of patients, results were expressed as medians and variations as interquartile range [IR]. Non-parametric statistical analyses were used: differences between two independent groups were determined by using the Mann Whitney U test with the Bonferroni correction for multiple comparisons; differences in more than two groups were determined by using the Kruskal Wallis one-way analysis of variance. Survival was defined as the interval between sampling date and date of death from cancer (cancer-specific survival). Patients alive and patients who died from non-cancer related causes were censored. Survival data were updated in February 2008. The survival probability was estimated by using the Kaplan-Meier method [30]. Single-variable survival analyses were done by using the Wilcoxon and log-rank tests and multivariate regression with the Cox's model [31]. The Cox's model analysis was written by coding as Boolean data all variables that reached a 0.20 p level based on the results of the univariate analysis. For each variable, the proportional hazard assumption was tested graphically. Survival was analyzed using the SAS software package.

## Results

### Serum HE4 in patients with NSCLC and benign lung disease

**Fig 1:**
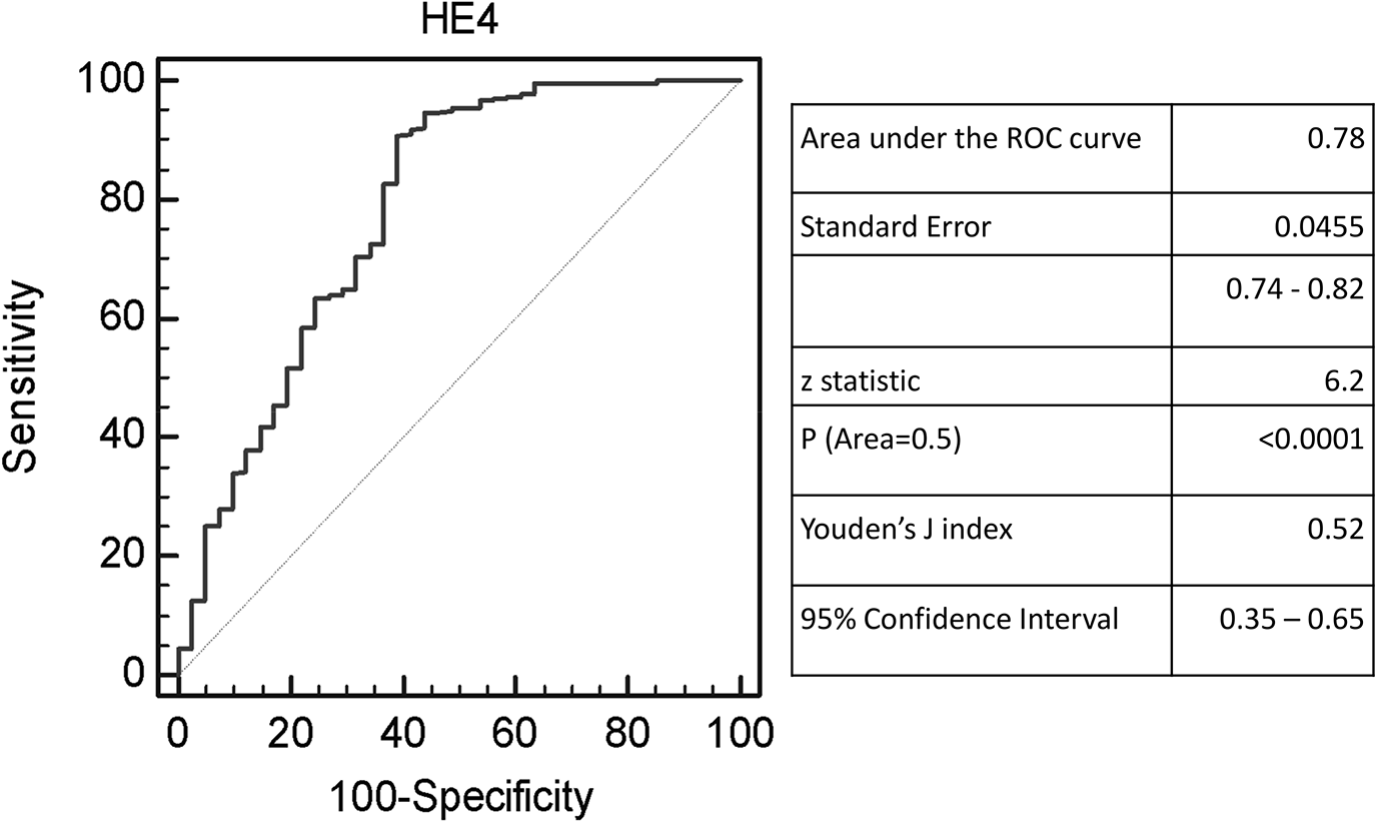
ROC curves constructed using the sensitivity–specificity relationship of HE4 to discriminate NSCLC patients and patients with a benign lung disease. Statistics using two-tailed Z-test.

Data on the population demography and disease characteristics are shown in Table 1. Serum HE4 ranged from 26 to 356 pmol/L (median = 50 pmol/L) in patients with benign lung disease (controls) and from 33 to 861 (median = 98 pmol/L) in patients with NSCLC (Table 2). The area under the ROC curve (AUC), which quantifies the overall ability of a marker to discriminate between controls and patients, was 0.78 (95% confidence interval (CI) [0.74–0.82]) and was statistically different from the non-discriminant bisector (z test: *P* <0.0001; Fig 1). Estimated HE4 sensitivity vs specificity according different cut-off is presented in supporting information (S1 Table). The Youden's index J (difference between true positive and false positive rate) was 0.52 and was associated with a cut-off value of 53 pmol/L (95% CI [50–67]), corresponding to a specificity of 91% and a sensitivity of 61%. This threshold showed the best sensitivity-specificity relationship in a diagnostic setting.

**Table 1:** Patients’ demographic and disease characteristics.

	*N = 346*
**Male gender**	311 (89.9%)
Mean (SD) age (years)	62.08 (10.9)
Performance status*	
0–1	182 (53.1%)
2–4	161 (46.9%)
Weight loss**	
≤5% of body mass	233 (71.0%)
>5 and ≤10%	58 (17.7%)
>10%	37 (11.3%)
**Histology**	
Squamous cell carcinoma	200 (57.8%)
Adenocarcinoma	94 (27.2%)
Large cell carcinoma and N.O.S. ^a^	52 (15.0%)
**Stage grouping+**	
Stage I-II	30 (8.7%)
Stage IIIa—IIIb	162 (46.8%)
Stage IV	154 (44.5%)
**TNM classification**	
1	16 (4.6%)
2	67 (19.4%)
3	78 (22.5%)
4	185 (53.5%)
**Nodal status**	
0	88 (25.4%)
1	13 (3.8%)
2	157 (45.4%)
3	88 (25.4%)
**Metastatic status**	
Yes	154 (44.4%)
Brain metastases‡	53 (15.3%)
Liver metastases	31 (9.0%)
Adrenal metastases	33 (9.5%)
Bone metastases	57 (16.5%)

* Three missing data.

** 18 missing data

+ according to the 6^th^ UICC classification

‡ the total of the different metastatic sites exceed 100% (not in the table) as metastases were frequently present in multiple sites.

^a^N.O.S.: not otherwise specified

**Table 2:** Serum HE4 distribution in patients with non-small cell lung cancer.

Serum HE4	*N = 346*
Mean (Standard deviation)	134 (121) pmol/L
Median (Min; Max)	98 (33–861) pmol/L
Percentile 2.5	45 pmol/L
Percentile 25	70 pmol/L
Percentile 75	147 pmol/L
Percentile 97.5	446 pmol/L
Number (%) of patients with serum HE4 >53 pmol/L	315 (91.0%)
Number (%) of patients with serum HE4 >140 pmol/L	95 (27.5%)

### Correlation with clinico-pathological characteristics

**Fig 2:**
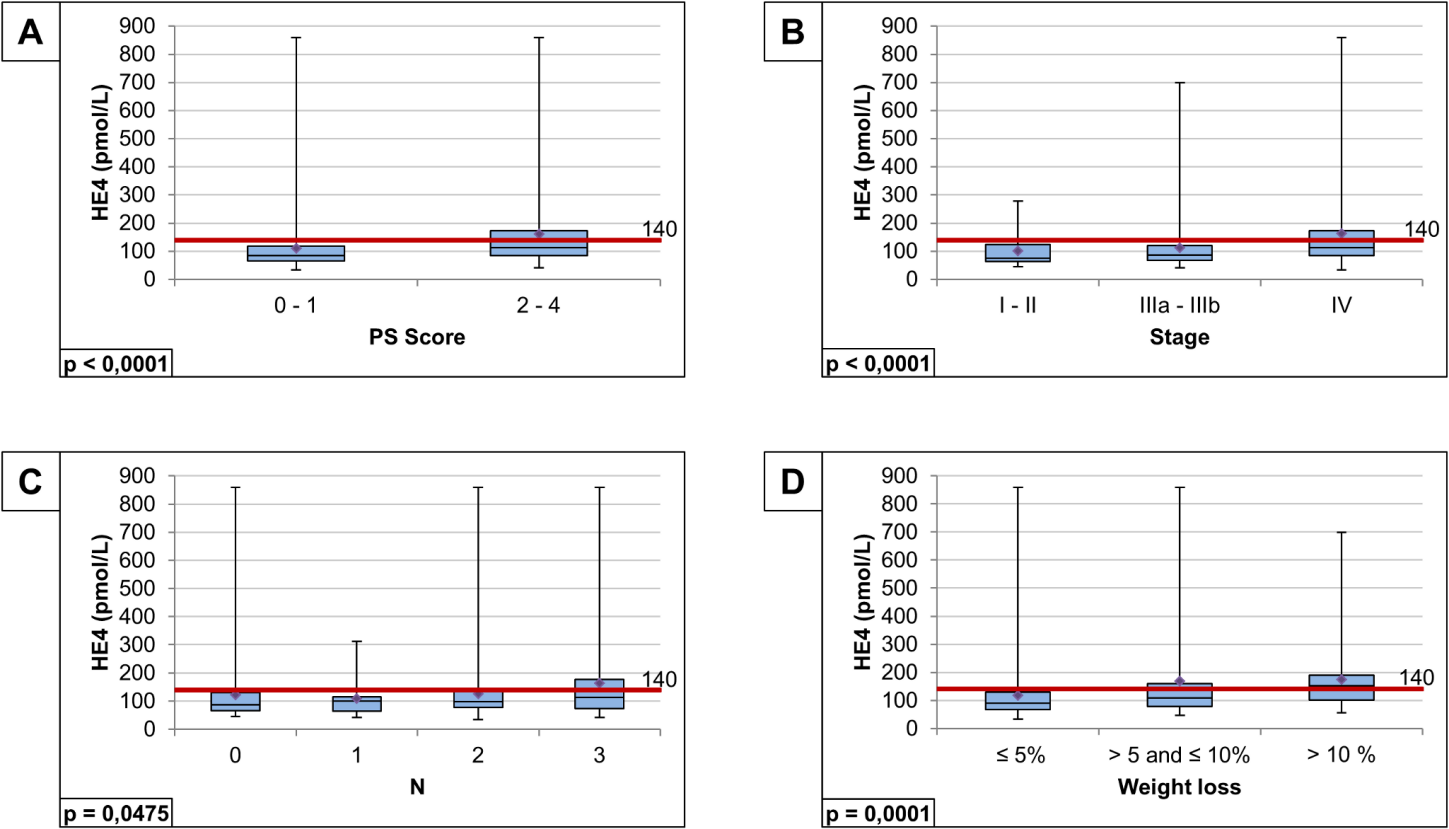
HE4 levels correlations with clinico-pathological characteristics. Serum HE4 levels significantly differed according to performance status (A), TNM stage (B) and nodal status (C) and weight loss (D)

Serum HE4 levels significantly differed according to performance status (p<0.0001), TNM stage (p<0.0001), nodal status (p = 0.047) and weight loss (p = 0.0001) (Fig 2). HE4 and CYFRA 21–1 serum levels were weakly correlated (Spearman’s coefficient r = 0.31, p<0.0001) No difference in HE4 values was found in the three histo-pathological subgroups (p = 0.1325, data not shown).

### HE4 and survival

**Fig 3:**
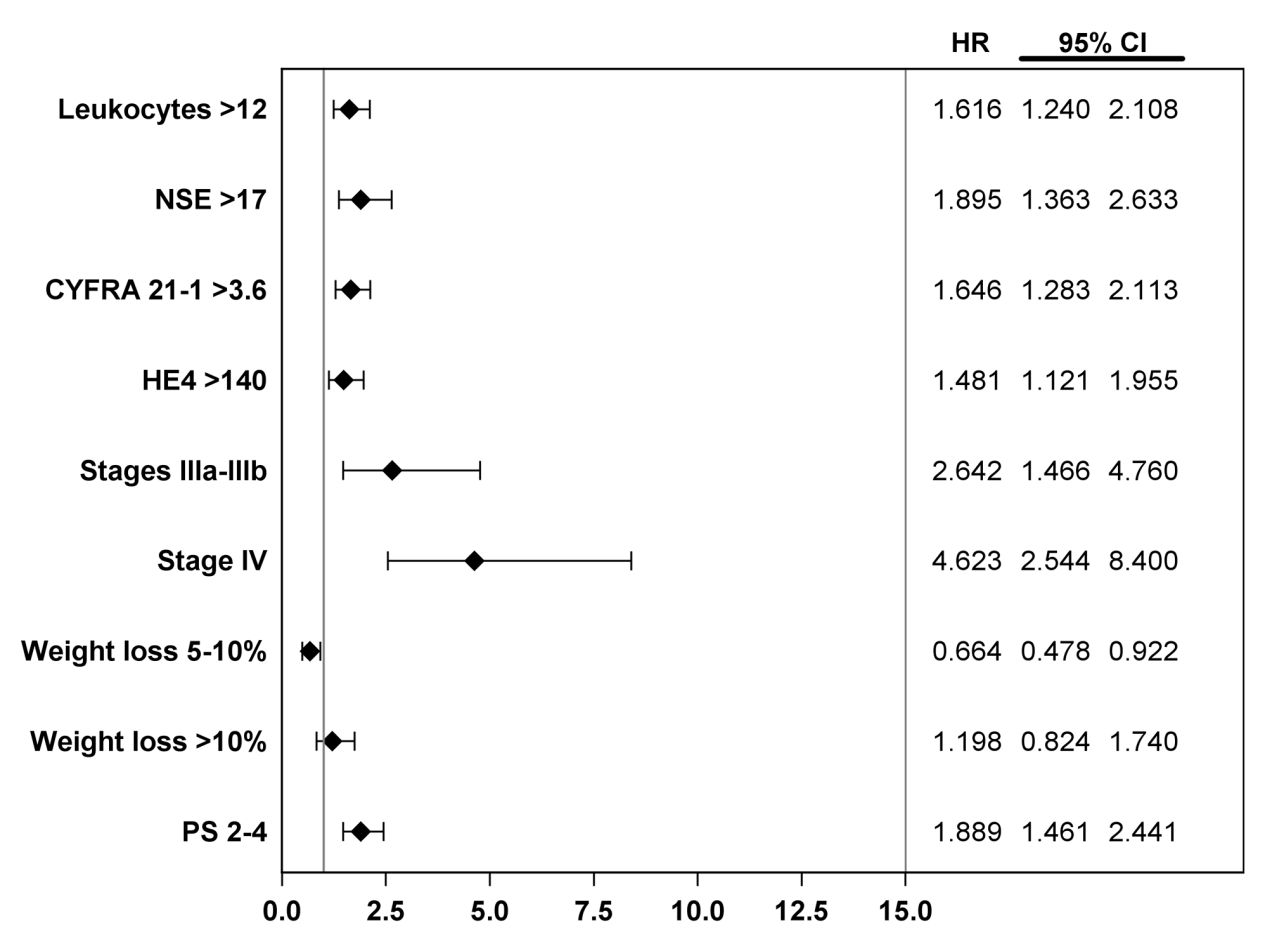
Forest plot of hazard-ratio for overall survival according independent prognostic determinants. HR: hazard ratio; NSE: neuron specific enolase; PS: performance status

**Fig 4:**
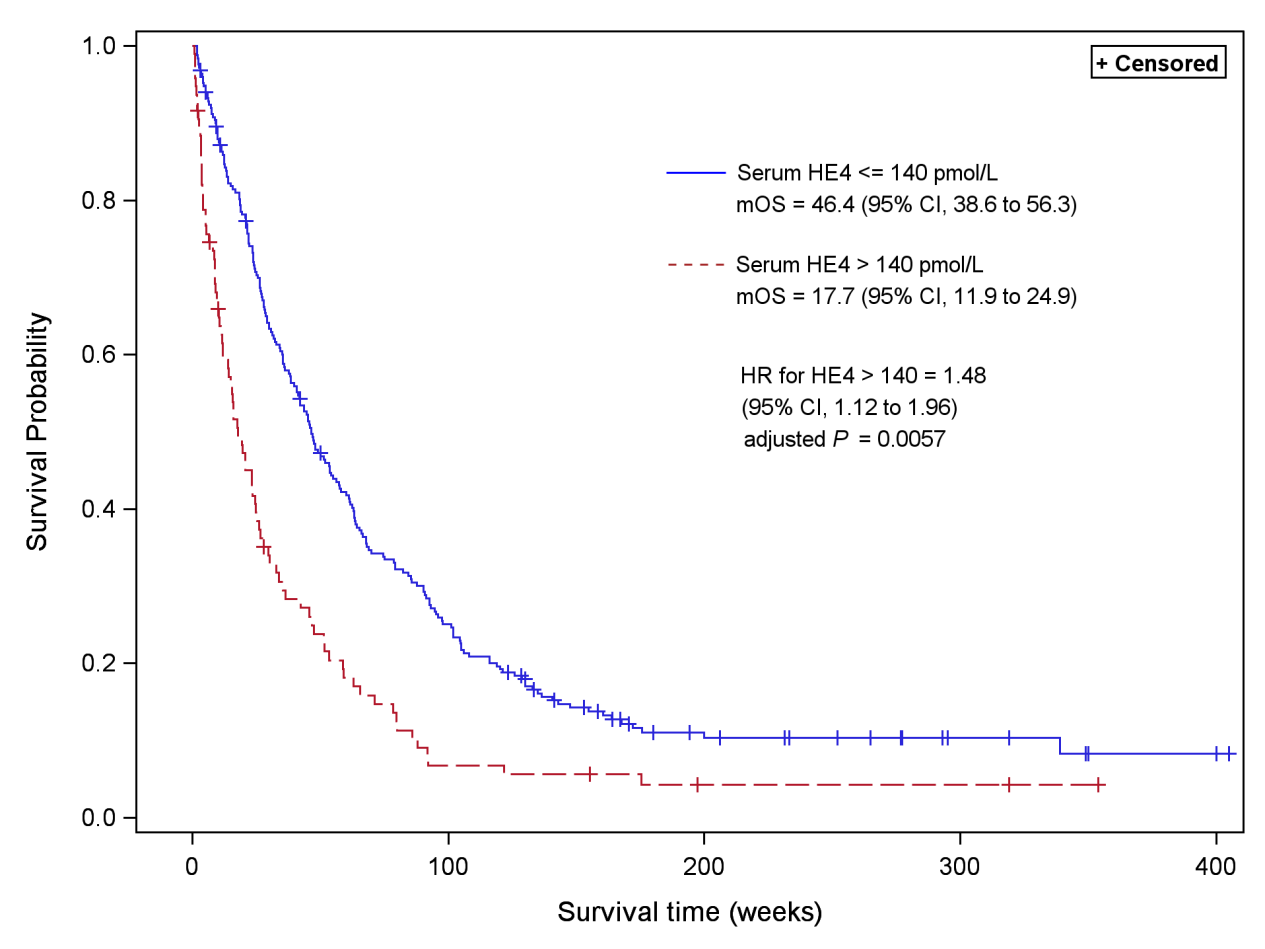
Probability of overall survival of NSCLC patients with < = 140 and > 140 pmol/L pre-treatment serum HE4 level. Kaplan Meier curves were constructed taking into account the whole population survival. mOS: median overall survival; CI: confidence interval

At the time of the analysis, with a minimum follow-up of four years and two months, 320 deaths (92%) had been recorded of which 302 (87.3%) were cancer-related. Seven patients (2.0%) were lost to follow-up and the median survival of the whole population was 36.4 weeks (30 to 45.6 weeks). TNM stage and nodal status were dichotomized (T1-2 versus T3-4 and N0-1 versus N2-3 respectively) to comply with the proportional hazard assumption. The biological and clinical features with a significant prognostic effect, based on univariate analyses, are listed in Table 3. Among them, there were many well-known prognostic determinants, including CYFRA 21–1 [36]. Serum HE4 was analyzed using two different thresholds: the 53 pmol/L diagnostic cut-off and 140 pmol/L, because this is the most widely used cut-off in the literature. The multivariate analysis is presented in supporting information S2 Table. In the Cox proportional hazard model, the independent determinants of a poor outcome were advanced stage, poor performance status, weight loss, high leukocytes count and high serum level of NSE, CYFRA 21–1 or HE4 (Fig 3). Patients with pre-treatment serum HE4 level higher than 140 pmol/L had a shorter overall survival when compared with patients with pre-treatment level ≤ 140 pmol/L. Specifically, the median survival was 17.7 weeks (95% CI, 11.9 to 24.9) for patients with serum HE4 higher than 140 pmol/L and 46.4 weeks (95% CI, 38.6 to 56.3) for patients with serum HE4 ≤ 140 pmol/L [HR: 1.48 (95% CI, 1.12 to 1.95) for high HE4; adjusted *P* = 0.0057] (Fig 4).

**Table 3:** Univariate analyses in patients with non-small cell lung cancer.

Variable	HR [95% CI]	P
**Serum HE4 (pmol/L)**		
≤53	1	
>53	1.54 [1.03; 2.31]	0.0352
**Serum HE4 (pmol/L)**		
≤140	1	
>140	1.96 [1.53; 2.53]	<0.0001
**Gender**		
Male	1	
Female	0.89 [0.62; 1.28]	0.5197
**Age**		
≤70 years	1	
>70 years	1.03 [0.79; 1.33]	0.8387
**Performance status**		
0–1	1	
2–4	2.18 [1.73; 2.74]	<0.0001
**Weight loss**		
≤5% (normal)	1	
>5 and ≤10%	1.13 [0.83; 1.53]	
>10%	1.84 [1.29; 2.626]	0.0036
**Histology**		
Squamous cell carcinoma	1	
Adenocarcinoma	0.73 [0.56; 0.96]	
Large cell carcinoma and N.O.S. ^a^	1.12 [0.81; 1.537]	0.0326
**TNM stage grouping**		
Stage I-II	1	
Stage IIIa—IIIb	3.32 [1.94; 5.69]	
Stage IV	6.73 [3.9; 11.61]	<0.0001
**Tumor status**		
1–2	1	
3–4	2 [1.51; 2.65]	<0.0001
**Nodal status**		
0–1	1	
2	2.32 [1.74; 3.11]	
3	2.94 [2.12; 4.06]	<0.0001
**Metastatic status**		
No	1	
Yes	2.41 [1.91; 3.05]	<0.0001
**Serum CYFRA 21–1 level (ng/mL)**		
≤3.6	1	
>3.6	1.99 [1.57; 2.50]	<0.0001
**Serum NSE level (ng/mL)**		
≤17	1	
>17	2.01 [1.49; 2.71]	<0.0001
**Serum sodium (mmol/L)**		
≥132	1	
<132	1.04 [0.53; 2.01]	0.9178
**Leukocytes (x 1000 /mL)**		
≤12	1	
>12	2 [1.56; 2.56]	<0.0001
**Albumin (g/L)**		
≥32	1	
<32	1.99 [1.4; 2.84]	0.0001

^a^N.O.S.: not otherwise specified

## Discussion

This study shows that high HE4 levels in the serum are related with poor NSCLC prognosis as patients with high serum level of HE4 before treatment had shorter survival. Well-known unfavorable clinical features, such as advanced stage, poor performance status and positive nodal status, were associated with high HE4 level. As these disease characteristics are also major prognostic determinants, one could hypothesize that HE4 acts as a surrogate variable when survival is analyzed. However, in the Cox model, high HE4 level was an independent prognostic determinant. Beside the fact that HE4 could segregate a sub-group of patients with shorter survival, its prognostic value suggests that HE4 secretion might play a role in NSCLC progression, like in ovarian cancer [37].

HE4 is a tumor marker mainly investigated in patients with ovarian cancer. This glycoprotein is also a component of the innate immune system of lung and respiratory tract and previous investigations suggested that serum HE4 could be a marker of NSCLC [27,28,29,38].

We compared HE4 level at diagnosis in patients with NSCLC or lung benign diseases. The ROC curve, constructed using the sensitivity–specificity relationship, indicated a good accuracy. At the optimal cut-off point of 53 pmol/L, determined with the Youden’s index J, serum HE4 presented a sensitivity of 91% and a specificity of 61%. Our results are slightly different from those reported in the study by Iwahori et al. [28] who found a ROC-AUC of 0.988. This discrepancy is explained by the different control populations used in the two studies (healthy population in the work by Iwahori et al and patients with benign pulmonary disease in our study). The ROC-AUC of 0.78 in our large population could be considered as more plausible in a diagnostic setting. The putative usefulness of serum HE4 for selecting candidates for low-dose computed-tomography screening programs deserves further study.

It has been previously suggested that serum HE4 sensitivity differs between genders [38]. The over-representation of male patients in our study (89.9%) precluded any confirmation of this observation. The proportion of SQC towards non-SQC in our study reflects the lung cancer taxonomy in non-selected European Caucasian population. Additional studies in other populations reflecting more accurately the current NSCLC epidemiology (i.e., the increasing incidence of adenocarcinoma and lung cancer in women) are needed. Attention should be paid to the influence of age and renal function on HE4 serum levels in future studies.

Our study prospectively analyzed biological and clinical variables in 346 patients with NSCLC referred to our institution with a minimum follow-up of four years and two months. These results strengthen the putative usefulness of this new prognostic marker and its relative place relative to key and currently used variables (i.e., performance status, TNM stage and other markers, such as CYFRA 21–1 and NSE). CYFRA 21–1, an extensively described NSCLC marker, was simultaneously evaluated in this study and high pre-treatment CYFRA 21–1 level was considered an independent prognostic marker. The congruent hazard ratios for high CYFRA 21–1 level in this work (1.646; 95% CI, 1.283–2.113) and in the larger population (1.41) of a study we published previously support the reliability of these survival analyses [39].

In conclusion, a high serum HE4 level at diagnosis is an independent determinant of poor prognosis in NSCLC.

## Supporting Information

S1 Table Estimated HE4 sensitivity vs specificity. (DOC)

S2 Table Multivariate analysis. (DOC)
