# Supplementary material for: Serum HE4: An Independent Prognostic Factor in Non-Small Cell Lung Cancer
Source: PLoS One. 2015 Jun 1;10(6):e0128836. doi: 10.1371/journal.pone.0128836 (PMC4452338; doi:10.1371/journal.pone.0128836)
Supplement: S1 Table — (DOC) [file pone.0128836.s001.doc]

Table S1. Estimated HE4 sensitivity vs specificity

| **Sensitivity (%)** | **Specificity (%)** | **95% CI^a^** | **HE4 (pmol/L)** |
| --- | --- | --- | --- |
| 80 | 63.4 | 46.3 to 75.6 | >66.9 |
| 90 | 61.0 | 43.9 to 75.3 | >57.7 |
| 95 | 51.2 | 31.0 to 65.8 | >50.2 |
| 97.5 | 39.0 | 21.8 to 53.7 | >45.0 |
| **Specificity (%)** | **Sensitivity (%)** | **95% CI^a^** | **HE4**  **(pmol/L)** |
| 80 | 51.7 | 32.7 to 72.7 | >97.3 |
| 90 | 34.0 | 12.3 to 56.3 | >122.0 |
| 95 | 25.0 | 3.9 to 41.8 | >146.0 |
| 97.5 | 12.6 | 2.2 to 32.9 | >202.4 |

^a^CI: Confidence Interval
