# Supplementary material for: Serum HE4: An Independent Prognostic Factor in Non-Small Cell Lung Cancer
Source: PLoS One. 2015 Jun 1;10(6):e0128836. doi: 10.1371/journal.pone.0128836 (PMC4452338; doi:10.1371/journal.pone.0128836)
Supplement: S2 Table — (DOC) [file pone.0128836.s002.doc]

Table S2. Multivariate analysis

|  | *HR [95% CI]^a^* | *p* | *Global p* |
| --- | --- | --- | --- |
| **Score PS** |  |  |  |
| 0-1 | 1 |  |  |
| 2-4 | 1.889 [1.461; 2.441] | <0.0001 | <0.0001 |
| **Weight loss** |  |  |  |
| ≤5% (normal) | 1 |  |  |
| >5 and ≤10% | 0.664 [0.478; 0.922] | 0.0146 |  |
| >10% | 1.198 [0.824; 1.740] | 0.3440 | 0.0184 |
| **Stage grouping** |  |  |  |
| Stage I-II | 1 |  |  |
| Stage IIIa - IIIb | 2.642 [1.466; 4.760] | 0.0012 |  |
| Stage IV | 4.623 [2.544; 8.400] | <0.0001 | <0.0001 |
| **HE4** |  |  |  |
| ≤140 | 1 |  |  |
| >140 | 1.481 [1.121; 1.955] | 0.0057 | 0.0057 |
| **CYFRA 21-1** |  |  |  |
| ≤3.6 | 1 |  |  |
| >3.6 | 1.646 [1.283; 2.113] | <0.0001 | <0.0001 |
| **NSE** |  |  |  |
| ≤17 | 1 |  |  |
| >17 | 1.895 [1.363; 2.633] | 0.0001 | 0.0001 |
| **Leukocytes** |  |  |  |
| ≤12 (normal) | 1 |  |  |
| >12 | 1.616 [1.240; 2.108] | 0.0004 | 0.0004 |

^a^CI: Confidence Interval
